# Tracking COVID-19 urban activity changes in the Middle East from nighttime lights

**DOI:** 10.1038/s41598-022-12211-7

**Published:** 2022-05-16

**Authors:** Eleanor C. Stokes, Miguel O. Román

**Affiliations:** 1 Earth from Space Institute, Universities Space Research Association, Columbia, MD 21046 USA; 2 Leidos, Reston, VA 20190 USA

**Keywords:** Energy and society, Energy and behaviour, Viral infection

## Abstract

In response to the COVID-19 pandemic, governments around the world have enacted widespread physical distancing measures to prevent and control virus transmission. Quantitative, spatially-disaggregated information about the population-scale shifts in activity that have resulted from these measures is extremely scarce, particularly for regions outside of Europe and the US. Public health institutions often must make decisions about control measures with limited region-specific data about how they will affect societal behavior, patterns of exposure, and infection outcomes. The Visible Infrared Imaging Radiometer Suite Day/Night Band (VIIRS DNB), a new-generation space-borne low-light imager, has the potential to track changes in human activity, but the capability has not yet been applied to a cross-country analysis of COVID-19 responses. Here, we examine multi-year (2015–2020) daily time-series data derived from NASA’s Black Marble VIIRS nighttime lights product (VNP46A2) covering 584 urban areas, in 17 countries in the Middle East to understand how communities have adhered to COVID-19 measures in the first 4 months of the pandemic. Nighttime lights capture the onset of national curfews and lockdowns well, but also expose the inconsistent response to control measures both across and within countries. In conflict-afflicted countries, low adherence to lockdowns and curfews was observed, highlighting the compound health and security threats that fragile states face. Our findings show how satellite measurements can aid in assessing the public response to physical distancing policies and the socio-cultural factors that shape their success, especially in fragile and data-sparse regions.

## Introduction

The Middle East faced unique challenges when responding to the onset of the COVID-19 pandemic. Several countries in the region entered the pandemic embroiled in political turmoil and conflict, with fractured governance, lack of transparency, and under-resourced public health systems, which complicated the response^1,2^. Furthermore, Ramadan, which began in April, posed the risk of becoming a multi-national COVID-19 “super-spreader” event^3,4^. Traditional religious practices and communal gatherings, rooted in solidarity and sharing, were at odds with public health guidance to privatize activity, close markets and public spaces, and cancel iftars, a traditional nighttime shared meal. In some countries, this tension resulted in governments loosening restrictions, caving to economic and religious pressure^5,6^, while in other countries strict curfews and physical distancing directives were bolstered^7^.

Physical distancing measures enacted to control COVID-19 transmission were varied across the region, both in their timing and their stringency, as were public responses to these measures. While there are now many databases that track the timing and details of COVID-19 government control measures (e.g. the Coronanet database^8^, HIT-COVID^9^, Oxford-19 Government Response Tracker^10^, etc.), there is not commiserate data on societal adherence—i.e., how the public altered their activity patterns in response to physical distancing measures.

Characterizing adherence is critical for forecasting the spread of viruses and for informing context-specific management of COVID-19 and future epidemics^11,12^. In previous studies, adherence has been captured through surveys^13–15^, news reports^16^, and through social media sentiment analysis^17^. While useful for understanding local factors that influence transmission behaviors, these data sources are not systematically collected, have limited geographic coverage, and are thus difficult to scale across space and time. They also most often measure public opinion towards physical distancing measures, which is important for understanding the motivations that influence activity patterns, but is different than capturing activity itself. Furthermore, these data are subject to selection bias since the users of media platforms are not necessarily representative of the whole population (e.g. due to differences in internet/phone access, technology use by age group or wealth, etc.).

**Figure 1:**
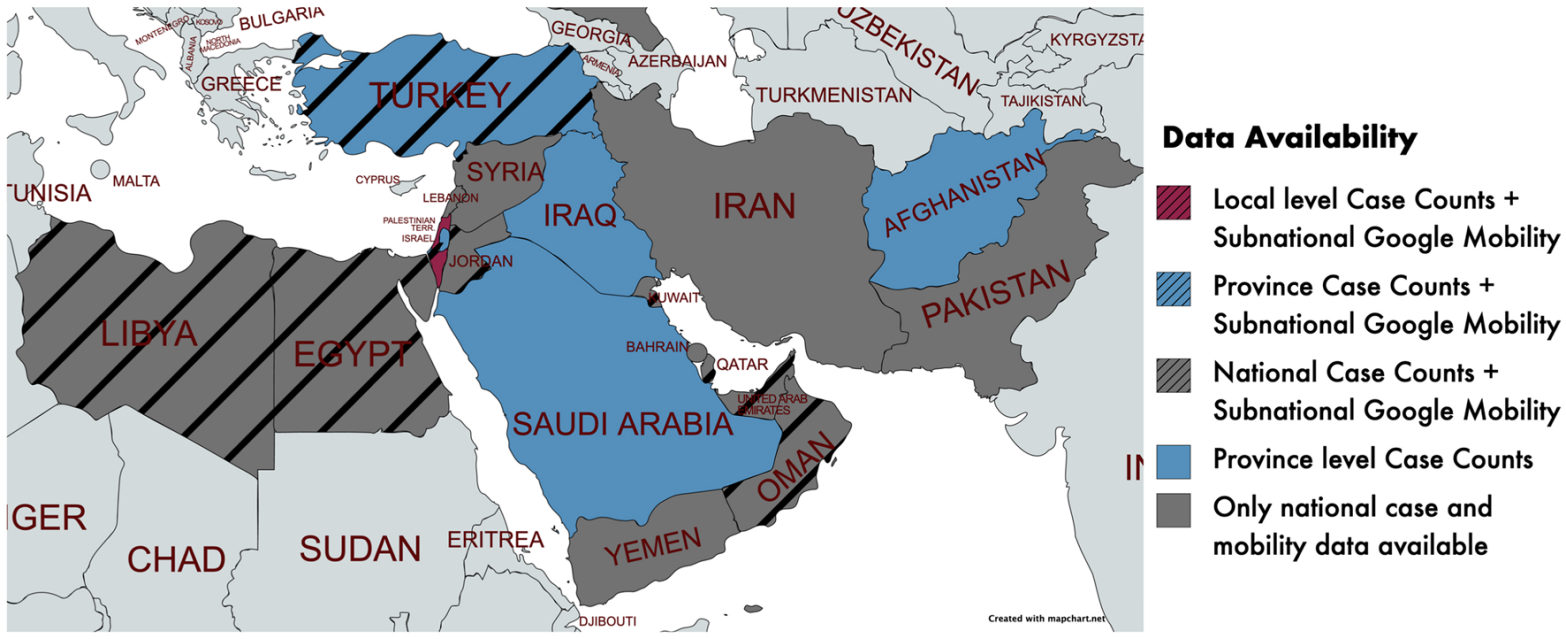
Map of countries in the study area, shaded to indicate data availability. Striped countries have sparse sub-national Google mobility data available, whereas non-striped countries have no sub-national Google mobility data. Countries in purple have urban-level COVID-19 case counts, ones in blue have provincial level case counts, while countries in dark grey only have national level COVID-19 case counts. Of our studied countries 6 had no sub-national data and only one had local level case count and mobility data. Map created using www.mapchart.net version 1.0.

Cell phone location data, collected by private companies, is the most common data source for tracking changes in societal activity patterns^11,12^. When the COVID-19 pandemic began, Google and Apple began releasing mobility data derived from their mapping products (Google and Apple Maps) that is usually kept private^18,19^. The mobility data tracks cumulative daily trips, by geography, using different modes of transportation to reach destinations like retail and recreation, groceries and pharmacies, parks, transit stations, workplaces, and residential areas. Google and Apple mobility data are available for thousands of cities, but coverage is biased towards North American and European regions (80% for Apple; 53% for Google), and there are substantial data gaps over parts of Africa, Asia, and the Middle East. The regions with the sparsest coverage by Google and Apple are the regions where transmission is the least understood, due to lack of resources, opaque data collection systems, or little testing of caseloads. In fact, 12 out of the 19 countries in the Middle East have no sub-national Google or Apple mobility data available for tracking changes in activity (Fig. 1). Where sub-national Middle Eastern mobility data does exist, it is usually aggregated to the province level, so activity patterns at town or city scales can not be differentiated.

Because of this data gap, understanding about societal adherence to COVID-19 physical distancing measures in developing regions remains limited. Earth-orbiting satellites, capable of global surveys at fine spatial scales, show potential for providing systematic measurements over data sparse regions. In particular, the Visible Infrared Imaging Radiometer Suite’s (VIIRS) day/night band (DNB), a new-generation space-borne nocturnal low-light sensor, is uniquely able to capture human activity from space^20^. The VIIRS-DNB measures nighttime lighting (NTL) from street lights, building windows, business districts, and vehicle headlights on a daily basis, characterizing the rhythms of human civilization, associated with holidays^21,22^, conflict^23,24^, and population movements^25,26^.

Recently, studies using simple differencing of monthly VIIRS-DNB composites observed that nightlights decreased in the first month of the pandemic across twenty global mega-cities^27^, and over China^28,29^ and India^30^. These studies indicate that the VIIRS-DNB may be capable of tracking the changes in human activity patterns associated with COVID-19, despite having an overpass around 1:30 am^31^. However, NTL time-series are not stationary^21,32^, so robust time-series analyses are needed that move beyond before-after snapshot comparisons of COVID’s impact. Furthermore, no studies have used NTL to explore the variation in responses to COVID-19 measures within and across countries, nor assessed their promise as a substitute or supplement for mobility over data-sparse regions.

Here, we assess the ability of NTL to track activity changes from COVID-19 physical distancing measures across 584 urban areas in 19 countries in the Middle East. We use openly-available nocturnal satellite imagery (NASA’s Black Marble VNP46A2, produced from the VIIRS-DNB on Suomi-NPP) to explore daily NTL changes in each urban area from before the start of the pandemic through June 25, 2020, a month after the end of Ramadan. Data processing techniques used to aggregate NTL data within urban areas, construct each urban NTL time-series, and control for quality observations are described in Methods: nightlights dataset. Time-series decomposition techniques are applied to measure how well NTL time-series synchronize with mobility data, and to explore the population-scale variation in activity change both across and within countries in the region. In addition, we compare Ramadan NTL patterns from 2015 to 2019 with those from 2020 to assess whether adherence to physical distancing measures changed when these directives were in conflict with traditional socio-cultural practices.

## Results

### Comparison with google mobility data

We used a time-series distance analysis between Black Marble NTL and Google mobility data on the 24 urban areas where there was an overlap in data coverage to measure time-series similarity. We also measured similarity at the national level in 8 countries that did not have urban-level Google data (see “Euclidean distance analysis” in “Methods” section).

**Figure 2:**
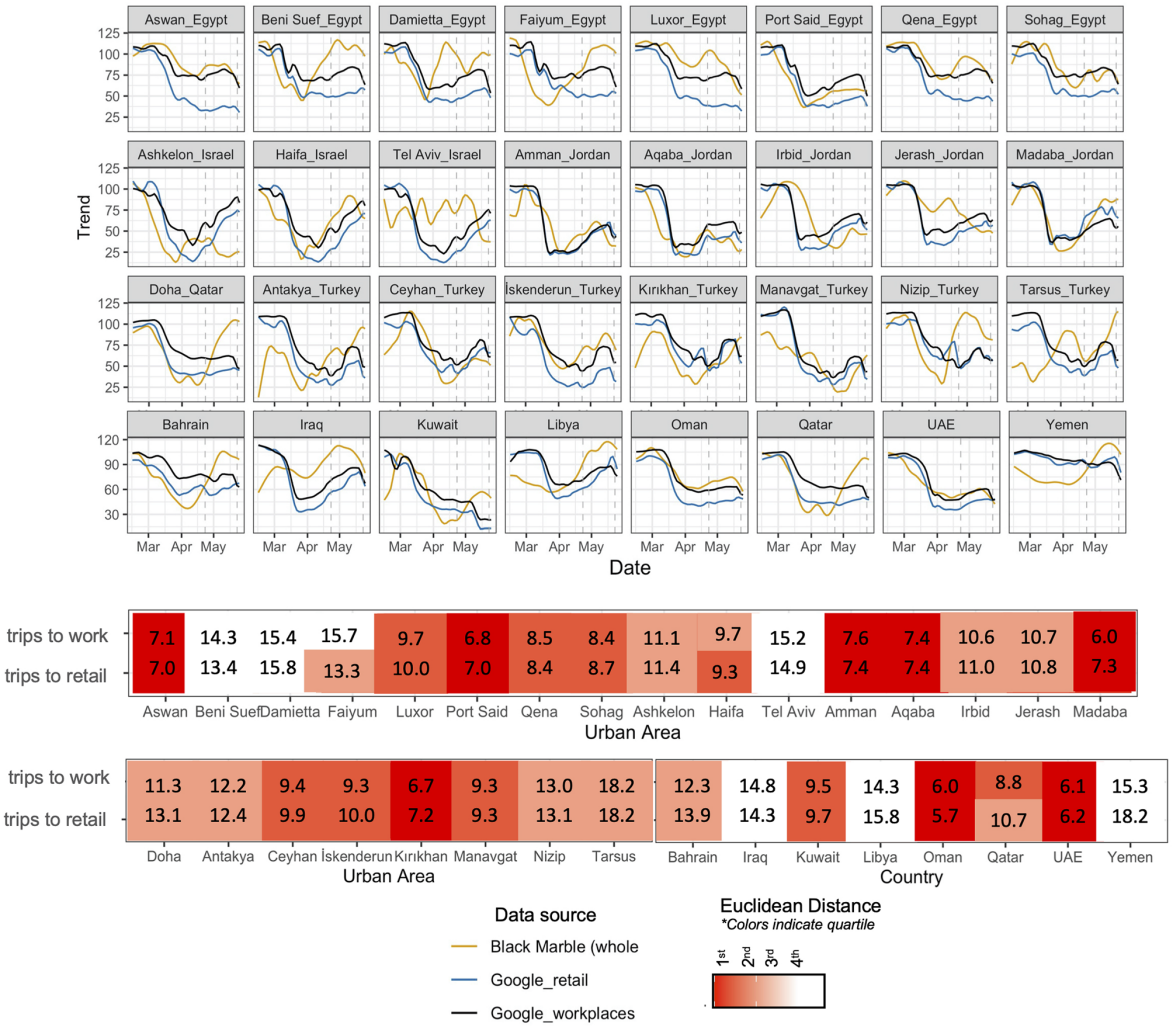
(Top) A visual comparison of Google mobility data time-series against Black Marble data (converted to the same scale) for urban areas in the Middle East. Ramadan period is framed by dashed grey lines). (Bottom) A euclidean distance measure between the Black Marble time series and Google mobility time series shows which cities and countries had high synchronicity between the datasets. Figure created using ggplot2 version 3.3.5^33^.

Mobility and NTL had similar temporal signatures in a large majority of urban areas and countries tested (Fig. 2). Both NTL and mobility data captured the initial decline in activity from COVID-19 control measures, which went in effect in late March. We also found that NTL time-series have a nearly equal similarity to both Google trips to workplaces and to retail.

For the majority of cities and countries mobility and NTL have noticeable synchrony, however there was wide variation in these patterns across and within different countries. The euclidean distance analysis confirmed the most consistent synchrony in Jordanian cities, as well as in the countries of Oman and the UAE. Within Turkey, Kirikhan, Iskenderun, Ceyhan, and Manavgat have Black Marble and mobility time series that closely track the same pattern, but in Tarsus there is little similarity. Similarly, in Haifa, Israel the two datasets are close, while in Tel Aviv they are not. At the national scale, we found that Iraq, Libya, and Yemen had the lowest synchrony—all countries which have been in conflict in recent years.

If NTL is to be used as a proxy for mobility data, future research must investigate the characteristics of a place that make this relationship reliably strong. Both trips and nighttime lights are affected by the physical distancing measures enacted during the pandemic, but they capture different, though related, kinds of behavior change. Within our sample of urban areas, one factor that seemed to affect the degree of correlation was the strength of a country’s Ramadan NTL signal. During Ramadan, daily fasting pushes meals and family gatherings later into the night, which disproportionately increases NTL. Black Marble and Google mobility time-series diverge during Ramadan (April 23–May 23 in 2020) for several of the Egyptian cities, for Nizip, Turkey and for countries like Bahrain, Qatar, Kuwait, and Yemen. Ramadan activities were disrupted by the pandemic, but Black Marble data still captured an increase over normal activity levels for many cities and countries that traditionally celebrate the holiday.

### Comparison with policy data enactment

Given the positive results from the mobility comparison analysis, we examined how well the timing of sharp decreases in Black Marble NTL corresponded to the enactment of COVID-19 restrictions. We iteratively identified the largest 15 sustained increases or decreases in radiance in the November 2018–June 2020 Black Marble time-series for each urban area, and the corresponding dates of change, magnitudes of change, and directions of change (“Time series disturbance analysis” in “Methods” section). We compared the dates of changes detected from NTL to dates of national curfews and lockdowns recorded in the publicly-available Coronanet government measures database^8^ for the countries in our study.

**Figure 3:**
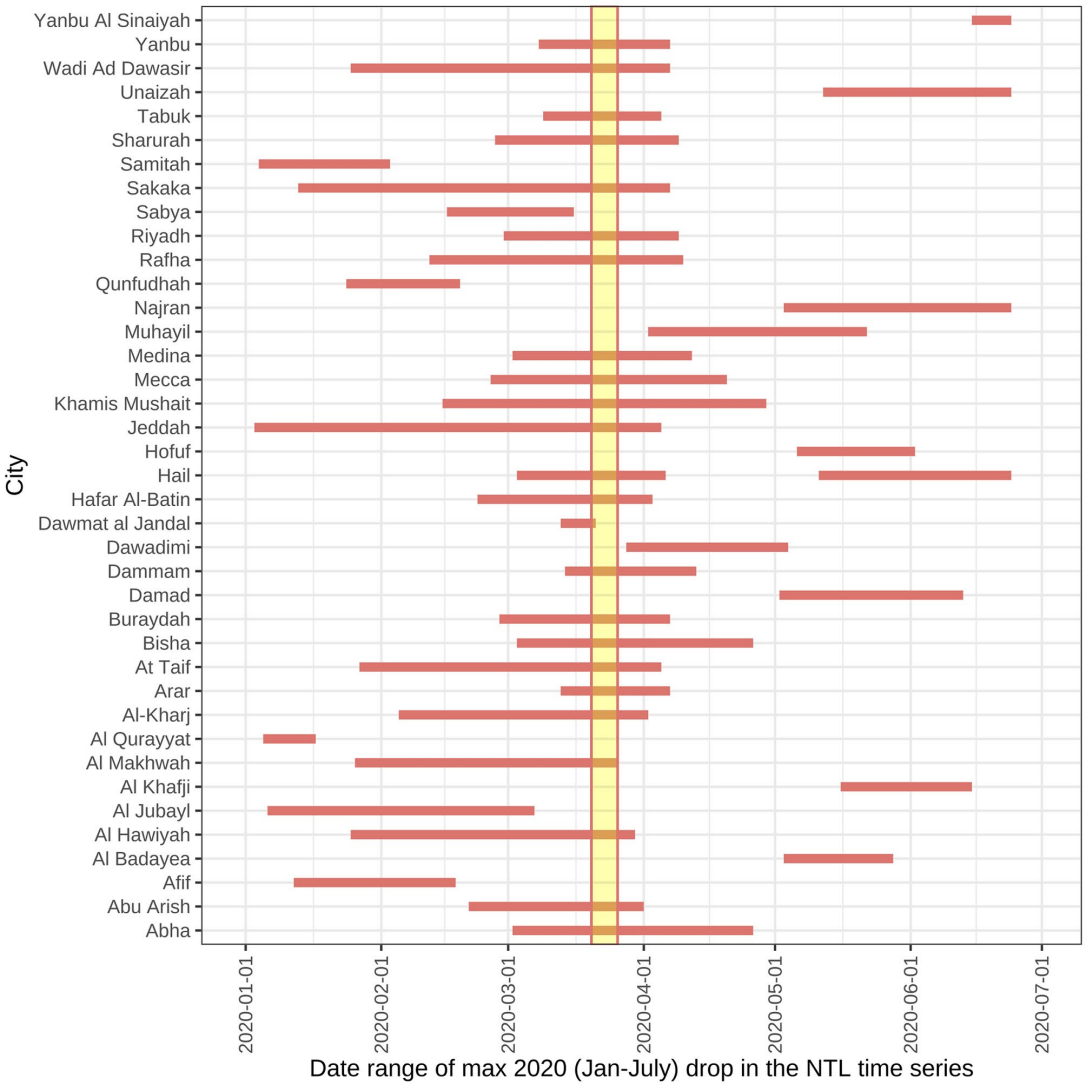
The date range of initialization of a national curfew in Saudi Arabia is depicted as a yellow rectangle, spanning a week, with the center falling on the day the lockdown began (3/23/20) for each urban areas in Saudi Arabia. The curfew initiation date is compared to the date range of the largest continuous decrease identified in the Black Marble NTL time series, represented as a horizontal pink line. For 24 of the 39 sampled Saudi Arabian urban areas (62%), the largest continuous decrease overlaps with the date range of curfew initialization. Figure created using ggplot2 version 3.3.5^33^.

In most urban areas, physical distancing policies caused a significant abrupt reduction in NTL and associated urban activities. For instance, the timing of lockdowns in late March corresponded to decreases in nighttime radiance of at least 5% in a large majority of urban areas in Saudi Arabia (82%) (Fig. 3). The figure also shows a second group of Saudi Arabian urban areas that had their largest reduction in NTL at the end of Ramadan (May 23–May 27), corresponding with the Eid al-Fitr. During this time, the Saudi government strengthened the national curfew, expanding it from 5pm-9am to a full 24 hours, to curtail any potential gatherings or celebrations that could have exacerbated the outbreak^34^. Of the 584 urban areas sampled, nearly 70% had a significant (i.e. top 15) decrease in radiance coinciding with the onset of physical distancing policies (Table S1).

Though predominant, national curfews and lockdowns did not result in a ubiquitous immediate decreases in urban activity in all countries. Only 37% of Turkish urban areas registered a drop in NTL bigger than 5%, when COVID-19 policies were put in place, Table S1). Countries in on-going conflicts (e.g. Iraq, Afghanistan, Libya, Yemen)—all of which are in the top 20 fragile states according to the Fragile States Index^35^—also had weak or inconsistent immediate nightlight decreases in response to national lockdown or curfews. Less than half of the urban areas sampled in these countries had a NTL decrease of greater than 5%.

### Long term national and urban variations in response to COVID-19 restrictions

Ramadan began almost two months after the first cases of COVID-19 in the Middle East, ushering in a new wave of COVID-19 control measures. Past work using Black Marble has shown that NTL radiance spikes for cities across the Muslim world during Ramadan, reflecting a shift in shopping, iftars, and community gatherings to later in the night^21,36^. To date there is little quantitative, trans-national information about how and where Ramadan activity changed in 2020, as compared to previous years, as a result of public health guidance to avoid these communal activities. Furthermore, little is known about whether the urban activity decreases detected at the onset of the pandemic in the Middle East were sustained for the duration policies were in place, or whether adherence waned.

To explore activity changes over the full first 4 months of the pandemic, we use an additive time-series decomposition to detrend the urban NTL time-series and separate seasonality. Time-series decomposition controls for simultaneous long-term non-COVID related changes occurring in the region, like electrification and urbanization, and allows for explicit examination of Ramadan holiday activity. After the decomposition, pre- and post-COVID-19 trend and seasonal radiance levels are compared, and the percent change for each urban area is computed (“Seasonal decomposition” in “Methods” section). We also classify the restrictiveness of government measures implemented in each country, from the start of the pandemic through June 25,2020, and during Ramadan, from April 23 to May 23, 2020, according to the COVID-19 Government Response Stringency Index^10^. Restriction strength is defined by the average of the stringency index over all dates included in each of the two time periods.

**Figure 4:**
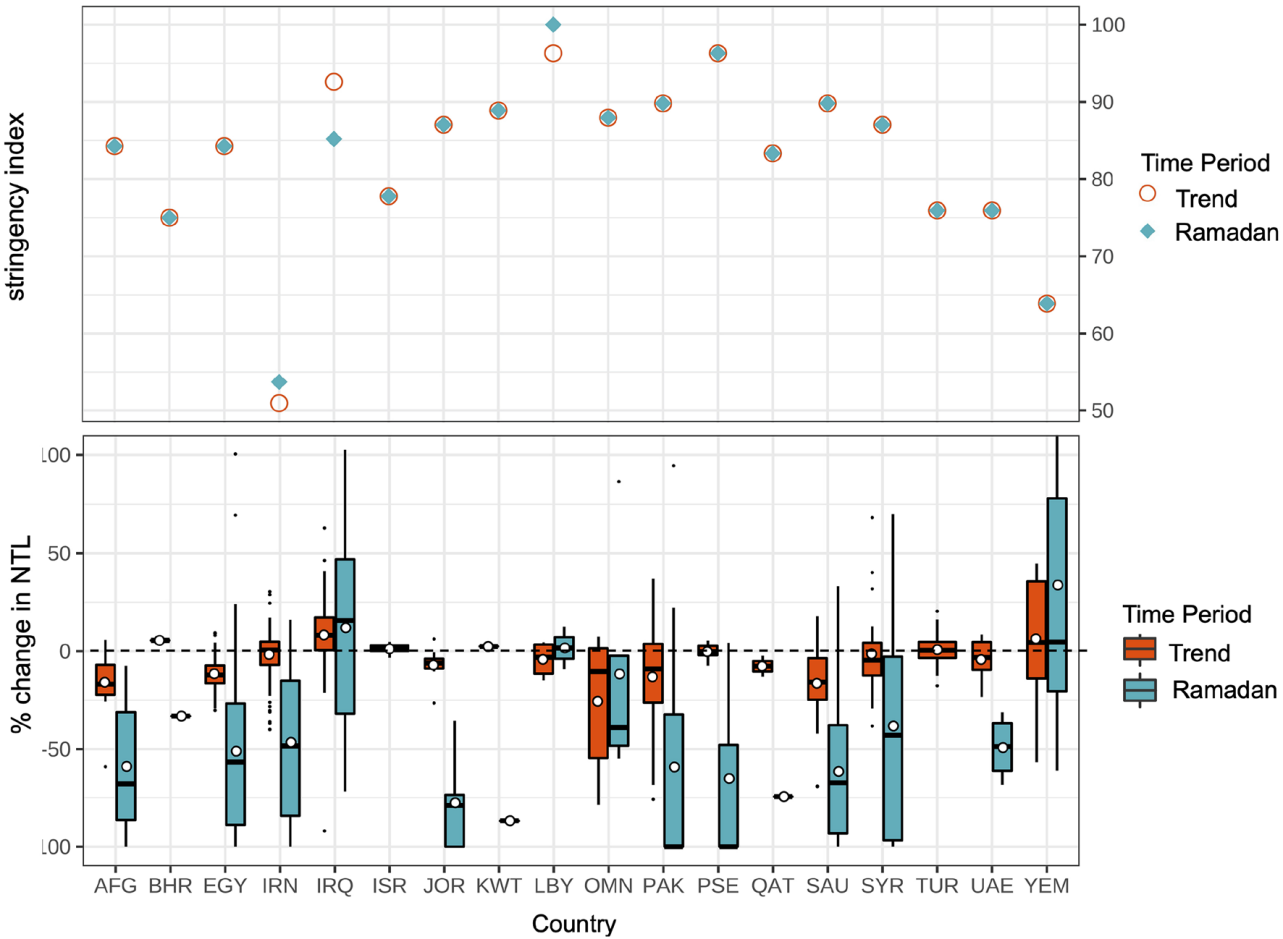
(Top) Restriction markers indicate the strength of national COVID-19 policies put into place during Ramadan and across the pandemic. These markers are compared with the boxplot below, showing the change between baseline NTL trend and Ramadan radiance. (Bottom) White circles indicate the mean of Ramadan and trend radiance percent change for the urban areas in each country. Figure created using ggplot2 version 3.3.5^33^.

How well do changes in activity correspond with differences in the strength of COVID-19 policies and regulations? Not surprisingly, we found that countries that enacted stringent control measures had the largest decreases in NTL radiance both during Ramadan and across the first 4 months of the pandemic (Fig. 4). Afghanistan, Egypt, Jordan, Oman, Pakistan, and Saudi Arabia, were all countries—with a restriction strength index greater than 85, reflecting strict daily curfews, cancellation of mass gatherings, and business closures. These countries also had the largest average trend (from − 7 to − 26%) and Ramadan (from − 11 to − 78%) decreases in NTL. Conversely, in the countries with the weakest restrictions—Iran, Turkey, Bahrain, UAE, and Yemen—we observed stable or increased trend NTL radiance in 2020, and smaller Ramadan decreases.

Countries with low societal adherence to control measures would theoretically show up as outliers or deviations in the relationship between policy stringency and NTL decrease. Iraq, Libya, Palestine, and Syria are four such outliers, all of which had restrictive control measures (an index above 85) but showed either positive or nearly no change in their average NTL trend radiance. For example, in Libya, where curfews and lockdowns were the most restrictive of all the countries in the Middle East, trend NTL radiance decreased by only − 4% and Ramadan NTL radiance increased 2% in 2020. These decreases are much less than would be expected by looking at peer Middle Eastern countries with restrictive policies.

**Figure 5:**
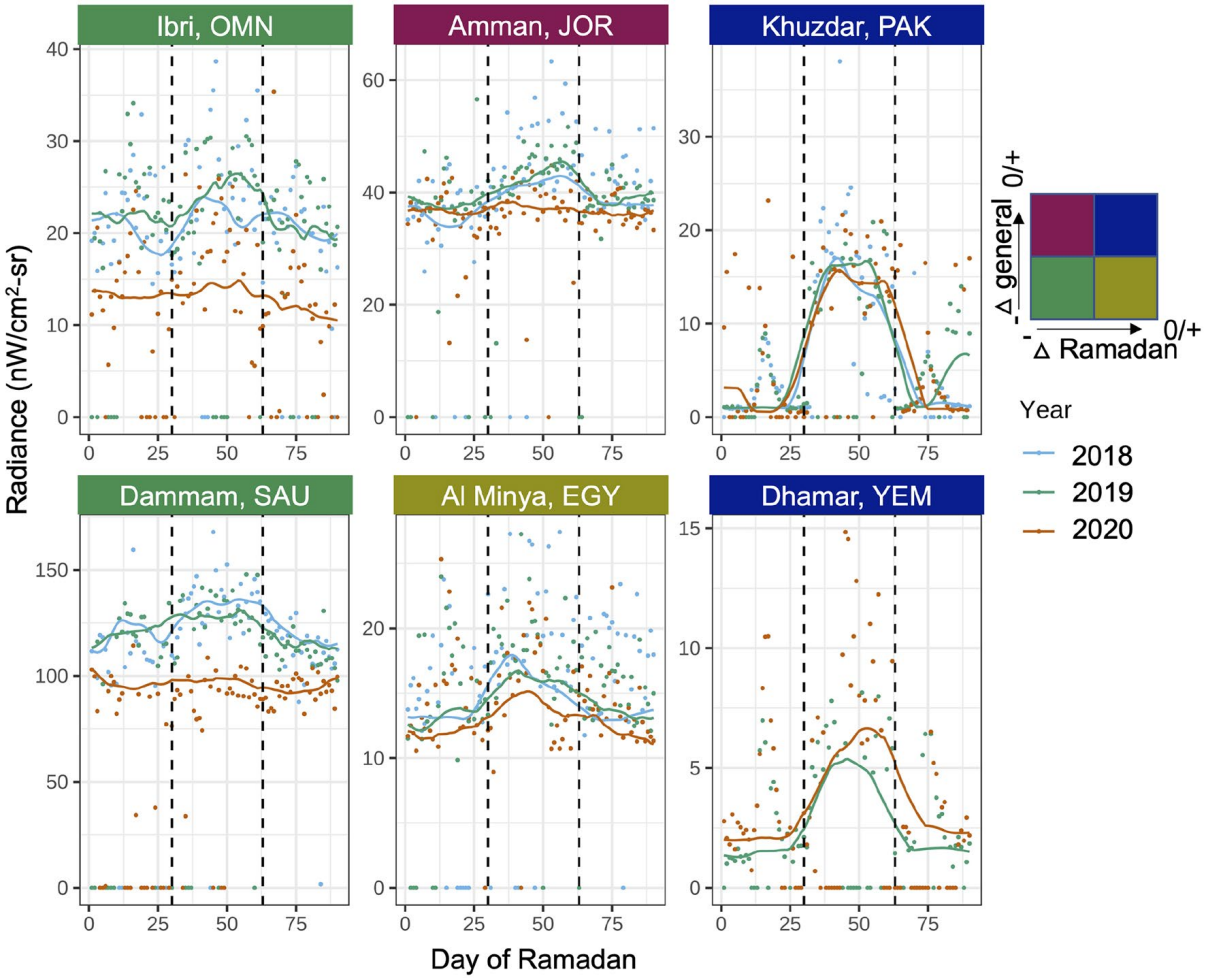
Historical and 2020 radiance for a sample of urban areas: (column 1), Ibri, Oman and Damman, Saudi Arabia are full responders, (column 2, top) Amman, Jordan is a Ramadan responder, (column 2, bottom) Al-Minya, Egypt exemplifies a seasonal city that is a general responder, (column 3) Khuzdar, Pakistan in the Balochistan province and Dhamar, Yeman are examples of full non-responders. The name boxes of urban areas are colored based on where they would fall on a two dimensional grid showing change in activity during Ramadan and generally during the pandemic. Figure created using ggplot2 version 3.3.5^33^.

Figure 4 also highlights the high heterogeneity within each country, both in the responses to Ramadan and control measures. Sub-nationally, four different typologies of urban COVID-19 responses emerged: (1) *full responders*: urban areas that had decreases in activity, both during Ramadan and generally during the pandemic (e.g. Ibri, Oman and Dammam, Saudi Arabia in Fig. 5), (2) *general responders*: urban areas where the NTL trend component decreased in response to the pandemic, but without a commiserate flattening in Ramadan seasonal activity (e.g. Al-Minya, Egypt in Fig. 5), (3) *Ramadan responders*: urban areas with a decrease in Ramadan activity, but little activity change in the post-COVID-19 era generally (e.g. Amman, Jordan in Fig. 5), (4) *full non-responders*: urban areas that showed little COVID-19 activity decrease during Ramadan and generally during the pandemic (e.g. Khuzdar, Pakistan and Dhamar, Yemen in Fig. 5). There are also urban areas that do not have a pronounced Ramadan signal pre-2020 (non-seasonal urban areas), which are classified solely according to their trend change.

**Figure 6:**
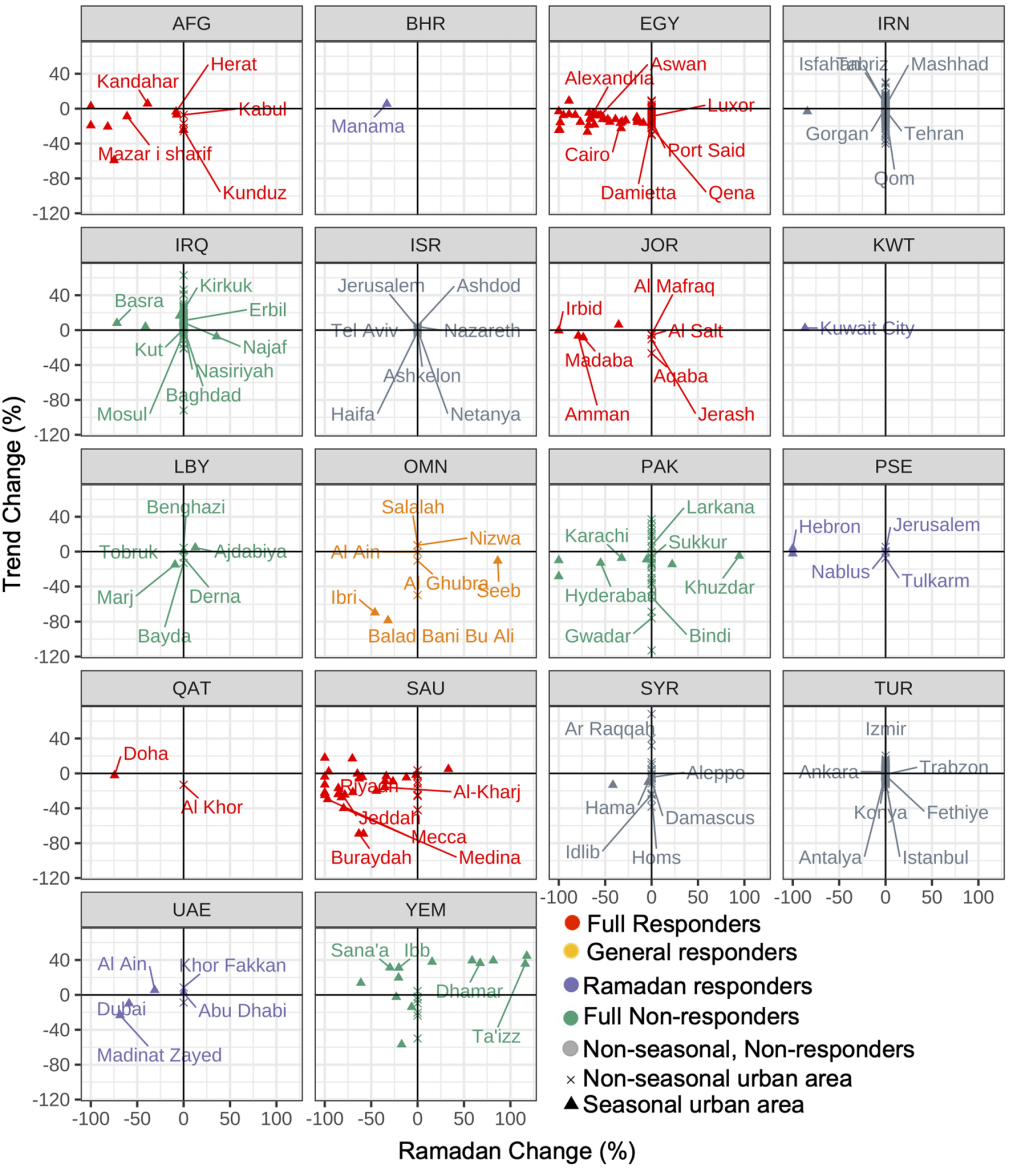
Percent change in Ramadan (seasonal) radiance is shown on the x-axis, while percent change in radiance generally during the remaining COVID-19 period (trend) is shown on the y axis. Country-plots are colored by the dominant typology of constituent urban areas. Seasonal urban areas (those with a consistent Ramadan increase, are represented as triangles, while non-seasonal urban areas are represented as an x. Figure created using ggplot2 version 3.3.5^33^.

These four typologies are represented in the four quadrants in Fig. 6. *Non-responders* (full and non-seasonal) occupy quadrant 1, with the post-COVID era having equal or increased trend and seasonal radiance as the pre-COVID era. Quadrant 2 and 4 host *Ramadan responders* and *general responders* respectively, with directionally opposite changes in seasonal and trend radiance. *Full responders* occupy quadrant 3, with both seasonal and trend decreases in radiance during the pandemic. The country plots in Fig. 6 are colored according to the predominant typology of the constituent urban areas.

Countries where a large majority of sampled urban areas were *full responders* included Afghanistan, Egypt, Jordan, and Saudi Arabia. In each of these countries, 85% or more of the urban areas had decreased Ramadan and general activity during the pandemic (Table S2). Qatar was also labelled a *full responder*, though its sample only included two urban areas, and Doha’s general decrease was quite mild (− 2%).

A second group of countries had consistent activity decreases (in 75% or more of the constituent urban areas) during Ramadan or generally, but not both. Three-quarters of Oman’s urban areas sustained decreased activity during the pandemic period (Table S2), but the decrease during Ramadan was less consistent, especially in the capital area of Muscat/Seeb. In Palestine, both Hebron and Jenin, the two sampled seasonal urban areas, showed strong reductions in Ramadan activity, but there was little commiserate change generally in the post-pandemic era, across urban areas (Fig. 6). Similarly, the UAE showed more consistent Ramadan decreases (67% of seasonal urban areas) than general decreases in activity (50% of urban areas).

*Non-responder* dominant countries were those with inconsistent changes amongst their urban areas. In the case of Pakistan, Turkey, and Iran, these inconsistent changes were the result of province-based approaches, Fig. 6. Turkey’s results (46% of urban areas with a general decrease) match its control measures, which were not national in extent, but limited to 31 selected cities^37,38^. In Pakistan, the urban areas of Karachi, Hyderabad, Kandhkot, and Mehar—all of which lie in the Sindh province—had large decreases in their Ramadan signal (− 71% on average). Conversely, in Khuzdar and Quetta, in the Balochistan province, NTL radiance levels stayed level or increased (+ 49% on average), as compared with pre-pandemic Ramadan activity. Our results show only 1/3 of Iran’s seasonal urban areas showed decreased Ramadan activity in 2020. On average, radiance in Iran decreased very little during the pandemic (by 1.5%), and only in less than half of the sampled urban areas.

A second category of *non-responder* countries were states in conflict. Iraq, Libya, Syria, and Yemen all had wide variation in their general and Ramadan responses. Yemen, in particular, was the only Middle Eastern country to celebrate Ramadan more in 2020 than in 2019, with an average increase in both general (6%) and Ramadan (9%) radiance (Table S3). In Libya and Iraq, only 1/3 of urban areas had reduced Ramadan activity in 2020, and less than half had decreased radiance generally during the the study period.

## Discussion

For directly transmitted infectious diseases, human to human contact shaped by daily activity patterns, plays a major role in shaping the dynamics of transmission in an epidemic. Increasingly, public health studies have pointed to the lack of social epidemiological data describing population dynamics as one of the biggest challenges to accurately characterizing the spread of pathogens^12,39,40^. In the case of COVID-19, physical distancing (e.g., avoiding travel and crowded public spaces, and limiting physical contact with others) has been an important strategy for containing transmission, though it is often not practiced uniformly across and within countries. There is little existing data that can capture the heterogeneous adherence to control measures across time and space, which are needed to forecast the effectiveness of these measures^11,12^.

Cell phone mobility data is increasingly being used to evaluate societal adherence to physical distancing measures^11,41^, but publicly-available data is patchy in coverage and nonexistent in many of the poorest, most fragile regions or in lower-resource settings, where use of smartphones is less common. It also often comes pre-aggregated so differences in local mobility patterns (towns or even neighborhoods) can not be examined. Currently when mobility data is not available, models are trained to predict activity patterns based on government policies alone^42^, a critical shortcoming in their applicability in regions where the divide between control measures and adherence to those measures is large.

Our results highlight the possibilities and limitations of NTL for providing context-specific information about how physical distancing control measures have changed human behavior on a societal scale. Unlike mobility data, global nightlights data is openly-available, collected continuously at 500 m resolution, and can be easily aggregated to describe changes in neighborhoods, cities, provinces, or countries, matching the spatial units of reported policy data, case and death count data. We find that NTL time-series during the first 4 months of the pandemic had high synchronicity with Google mobility data during the same time period, though the degree of synchoroncity varied across regions. This indicates that NTL can act as complement for mobility data when there is sparse coverage or a substitute when there is no availability. NTL also corresponded well with the timing and severity of governmental measures. Drops in radiance in the NTL time-series matched the onset of national control measures in 70% of the sampled cities, and stronger measures were associated with larger decreases in radiance. This confirms that the activities captured in NTL dynamics (e.g. business operations, traffic) indirectly capture the transmission activities that control measures target (e.g. mixing, human to human contact).

Equally important are differences between NTL time-series and mobility data, and the limitations of its use. First, while NTL has global spatial coverage and higher spatial resolution, it is more limited temporally. Cell phones track activity throughout the day, but existing Lower Earth Orbiting (LEO) satellites, like those that collect NTL data, overpass only once per night. Second, unlike trip destinations, the different activities that contribute to nightlights cannot be easily differentiated. NTL is primarily associated with downtown centers, commercial areas, manufacturing, public service areas, street lights, and transportation^43–45^, but how much each of these sectors contributes to nightlights radiance is variable from city to city, and across countries, making it difficult to identify or compare the causes of decreased urban activity. Furthermore, some sectors are largely invisible to nightlights data—e.g. communal gatherings in residential areas would not be captured even though they may drive increased transmission. Third, as with mobility, NTL dynamics are impacted by several socio-cultural and technical processes that are not related to COVID-19 transmission—e.g. conflict, urbanization, electricity instability and electrification. To measure adherence reliably, care must be taken to control for these processes and to establish a reliable baseline. Hence, the approach used here is not a replacement for cell phone data or surveys altogether, but is complementary, and may particularly benefit data sparse regions.

In parts of the Middle East, there is little understanding of numbers of cases, or even deaths, since such statistics are dependent on testing capacity and reporting policies. Recent studies estimate that only 1.25% of COVID-19 deaths in Damascus^42^ and 7.7% of deaths in Egypt have been reported^46^, spotlighting the Middle East as a region where COVID-19 transmission rates, and the activities that impact them, have been largely untracked. Activity patterns derived from nightlights provide insights into how the Middle East responded to control measures meant to slow the transmission of COVID-19, during Ramadan, and more generally across the first four months of the pandemic.

Our results show, on average, NTL decreases tracked restriction stringency for most countries in the Middle East. Not surprisingly, in countries with lenient restrictions (e.g. Yemen, Turkey), we found little change in activity between pre-pandemic years and 2020. The inverse was true for countries with restrictive policies (e.g. Jordan, Saudi Arabia). Outliers included Iraq, which saw an average increase in Ramadan activity over 2020 despite a national curfew, and Libya, Palestine, and Syria which all had restrictive control measures, but little NTL change. Within countries, we identified a highly heterogeneous response to COVID-19 restrictions, apparent in the degree of dispersion in the scatterplots in Fig. 6 and the standard deviations in Table S2, calling into question whether nationally-aggregated data on mobility or activity is even meaningful for local disease transmission forecasts.

Changes in nightlights are the result of behavioral changes influenced by both top-down policy, but also the values and decision-making apparatus of civil society. In some cases, like in Turkey, Pakistan, and Iran, the heterogeneity in responses reflects the balkanized application of top-down control measures across provinces. Turkey stood out from the rest of the region by not ordering a full national lockdown or curfew until April 2021^47^. The national government in Pakistan, under pressure both economically and from religious leaders, is widely considered to have had an inconsistent response early in the pandemic, sending mixed messaging and misinformation, backtracking on Ramadan restrictions, and downplaying the pandemic’s health risks^16,48^. This left many of the provinces to enact their own measures. Sindh province, which is run by the national government’s opposition party, took an early position on the pandemic in mid-March, ordering all public transport, markets, offices, shopping malls, restaurants, and public areas to be shut down^49^. These restrictions were consistently extended through the Ramadan holiday^50^. We observed significantly larger reductions in activity in Sindh urban areas during Ramadan than in non-Sindh urban areas. Similarly, Iran’s national government reversed proposed restrictions during Ramadan to appease conservative religious clerics^51^. The information apparatus in Iran was been widely regarded as unreliable^52^, which may have stifled activity decreases that otherwise would have been initiated by individuals or civil society.

In other cases, highly heterogeneous responses may reflect differences in the reaction of civil society to control measures. Our results expose a significant divide between control measures and activity changes in conflict countries. Fragile and conflict-afflicted countries, such as Syria, Iraq, Libya, and Yemen, had weak responses to COVID-19 restrictions and the lowest, least consistent decreases in nightlights for both Ramadan and generally. Yemen was the most lenient country in the Middle East, with few control measures in place. In contrast, Syria, Iraq, and Libya closed borders and suspended flights, shut down schools, imposed curfews, banned public gatherings, implemented mandatory lockdowns, and imposed strict social distancing, however NTL records indicate that little changed in the way of urban activity.

There is little available ground-truth data about changes in activity patterns in fragile states, however the data that exists validates the NTL records. Crowd-sourced photo submissions from Yemen, have indicated that markets in larger cities (e.g. Sana’a and Ibb) appear to be as busy as they were before COVID-19 restrictions were put in place^53^. Journalistic accounts describe Yemen as one of the only Muslim countries to celebrate Ramadan as usual in 2020^54^. In phone surveys conducted by the International Organization for Migration in Iraq in April and June, a majority of respondents said half or less of the population stays at home during curfews^55^.

Three hypotheses exist for why countries in conflict had smaller or inconsistent changes in activity, despite strict curfews. First, conflict zones often already have existing curfews in place and limits on freedom of movement and assembly, making COVID-19-specific behavior change less significant from baseline activity levels^56,57^. Second, residents living in fragile states must often prioritize safety, security, and economic concerns over complying with public health restrictions. In Iraq, a considerable percentage of the population resisted COVID-19 restrictions on movement, because of the need to protect their livelihoods^58^. In Syria, restrictions were reversed out of fear of economic collapse^59^. Across the Middle East, armed conflict increased during governmental COVID-19 lockdowns, presenting residents with a security-public health compound threat^60^. Third, given the fractured governance and public distrust of policing in conflict zones, there may be fewer resources available for education and enforcement of public health measures^61,62^. In Iraq and Libya, strict national restrictions were in place, but some studies document that the government and police forces had trouble convincing citizens to comply^63,64^.

Though the use of nighttime lights (NTL) to study activity changes requires some background understanding of the specific social contexts where it is applied, these preliminary results, along with the global coverage and open availability, make NTL data a promising resource for public health applications. Like many developing countries, most of the Middle East currently has vaccination rates below 30%^65^, making physical distancing measures one of the few available strategies for controlling virus spread. We suggest that the approach operationalized here may be used to adapt epidemiological models to a local context, sharpening COVID-19 transmission forecasts in developing countries, fragile-states, and regions with sparse mobility data. If successful, the approach may be expanded to other infectious diseases, to other locations (e.g. to examine traffic on roads that connect urban areas), where dynamic measures of human populations are needed to understand outbreaks.

While the VIIRS-DNB was designed for weather and environmental monitoring, the demonstrated value of satellite NTL data for the benefit of public health management opens up new discussions about the role Earth Observation could play to address pressing social challenges traditionally outside of the purview of Earth Science. More frequent NTL observations, captured across the night, is one investment that would have immediate public health benefits^31^. Multi-spectral nighttime platforms would also help to disaggregate the causes and sectors contributing to changes in urban activity. Apart from transforming control strategies for transmissible diseases, dynamic measures of human movement are also essential for immunization campaigns, disease surveillance, and improved response to disasters and outbreaks^66–68^. Providing actionable information to decision-makers about the nature and variety of shifts in human activities would allow for better understanding of their impact on disease and health.

## Methods

### Nightlights dataset

We used the 500 m daily NASA Black Marble nighttime lights product (VNP46A2, openly-available to the public at https://ladsweb.modaps.eosdis.nasa.gov/missions-and-measurements/products/VNP46A2/) to create a 5 year time series of nocturnal radiance observations over 7 tiles covering the Middle East. The Black Marble retrieval algorithm utilizes all high-quality cloud-free and snow-free radiances captured from the VIIRS-VIIRS-DNB sensor on-board the Suomi-NPP satellite, and corrects for atmospheric-, terrain-, vegetation-, lunar-, and stray light effects^69^. Image dates included the six months around Ramadan for 2015–2018 (3/18/15–9/15/15; 3/9/16–9/3/16; 2/26/17–8/24/17; 2/15/18–8/13/18) and 11/7/18–6/25/20.

We used three layers of quality control to create reliable urban nightlight time-series. First, we filtered only high-quality pixel-level observations (QA flag 0 or 1) within each urban area to be averaged each day, for each year. Urban boundaries were defined by the Global Human Settlement Functional Urban Areas (GHS-FUA) dataset^70^, which delineates the commuting areas of urban centers globally in 2015, based on objective characteristics like travel time to central business districts, population and population density, and country GDP.

Second, we filtered out low-quality daily urban radiance averages using a majority quality flag. The majority quality flag was based on the percentage of pixels in the urban area that are high quality (QA flag 0 or 1), low quality (QA flag 2), or no data (QA flag 255). If the majority pixels in the urban area were low quality or no data, that date’s urban average was excluded and the radiance value was interpolated. A 14 day rolling window was then used to smooth each of the urban time series and remove spurious measurements. The derived time-series for this study are included in the Supplementary Information (SI Dataset S1).

Finally, we filtered for reliable *urban time-series* based on the number and distribution of high quality observations. We created quality assessment summary statistics *E* and $\varepsilon$ for each time-series, where *E* is the proportion of high quality observations over the entire assessment period, and $\varepsilon$ is the proportion of rolling 7 day weeks with at least one high quality estimate. *E* and $\varepsilon$ are calculated as:

$$E=\frac{\sum \limits _{i=0}^n |w_i<2 |}{n} {\text{ and }} \,\varepsilon =\frac{\sum \limits _{i=0}^{n-7} ||{(w_i...w_{i+7})}<2 |>1 |}{n},$$

where $w_{i} \in \{w_{0}...w_{n}$} denotes the quality flag for each daily radiance observation of each urban area.

Urban areas with $E< 0.08$ or $\varepsilon < 0.7$ were excluded. These thresholds were tuned to balance the trade-off between keeping a high proportion of urban areas in the sample (65%) while ensuring the the time-series is stable, complete, and without multiple week gaps. 582 urban areas in 19 countries were included in the final sample.

### Euclidean distance analysis

To compare the similarity between nightlights signal and mobility signals, we first identified the overlapping subset of cities and countries included in both the Black Marble sample and the Google mobility dataset release. Metropolitan area data was only available for Egypt, Israel, Jordan, Turkey, and Qatar. On top of the quality filtering ($E< 0.08$ and $\varepsilon < 0.7$) for the full temporal range, we also specifically filtered for high quality Black Marble data in the first half of 2020, since that was the coverage of the Google mobility time-series. Twenty-four shared urban areas, with high-quality complete data records, were identified in these countries. We also included 8 other countries that had Google mobility data available at the national level.

Google’s mobility data is provided originally as % change from baseline, with baseline represented as 0. For the figures, we convert google mobility time series, so that baseline is represented as 100, and then scale the Black marble time series data, from 1/01/2020–6/25/20, to match the range of the Google mobility data. After visually checking for local stationarity in the Black Marble time-series, we use a 7 day moving average to reduce noise and eliminate weekday vs weekend periodicity.

To measure the distance between Black Marble and Google mobility datasets, we chose a euclidean distance measure^71^. Euclidean distance measures are often rejected for other time-invariant distance measures like dynamic time warping, however for this study we wanted to capture variations in the timing of drops and peaks between mobility and NTL datasets. We z-normalize all time-series to make them scale invariant and report out the euclidean distance as a measure of similarity between NTL and mobility in each city/country in Fig. 2. We also report the distance, excluding the Ramadan period in the SI (Table S4) to help identify cities where Ramadan is the main contributor to NTL and mobility differences. Urban areas that had missing measurements in the google mobility data were excluded from the analysis, as were Ramadan measurements since Ramadan activities are only captured by the Black Marble data.

### Time series disturbance analysis

To identify major breaks and decreases in NTL, we first apply a 30 day moving average to the 11/7/18–6/25/20 Black Marble time-series to smooth out all minor perturbations and identify the most prominent features. Using run length encoding, we divide each time-series into segments that continuously rise or fall, and require that the segments are at minimum 5 days in length, to rule out short-term anomalies like power outages. The largest 15 sustained changes are recorded, along with the corresponding dates of change, magnitudes of change, and directions of change for each urban area. Sustained increases are discarded, to leave a dataset with the largest decreases for each urban area.

This dataset is merged with information about the date range of national lockdowns and curfews for each country included in the publicly-available Coronanet government measures database^8^. Yemen, Palestine, Pakistan, Bahrain, Iran, and Qatar had no data, so information about measures in these countries were sourced from news articles. In Pakistan, provinces were primarily responsible for early lockdowns, which varied, so the authors chose lockdown dates for the Sindh province, matching the location of the majority of urban areas in our sample. Yemen and Bahrain avoided curfews, so for these countries, the implementation of restrictions was used as the policy date. Iraq and Lebanon both started with partial lockdowns and escalated to full lockdowns, so both dates were included—the date range of initialization and date range of intensification.

We identify the overlap between COVID-19 measures in each country and identified sustained COVID-19 decreases in each urban area. Table S1 reports the the percent of overlap: urban areas that had a 1%, 5% and maximum NTL decrease while COVID-19 measures were implemented.

### Seasonal decomposition

We use an additive STL Loess time-series decomposition to disaggregate changes in radiance from COVID-19 during Ramadan and generally throughout the pandemic period. The decomposition splits the time series into trend, seasonal, and remainder components. We create a discontinuous time series to input into the decomposition created from the 90 days before during and after Ramadan each year from 2015 to 2019 (540 days total, 6 periods). The trend is computed as a step function from the average of the data for each year. After detrending, the seasonal component is extracted by taking the median radiance value of each of the 90 days across the first 5 years. The remainder is the leftover when the trend and seasonal component (repeated each year) is subtracted from the original time-series. The decomposed components and the seasonal and trend percent change are listed for each urban area in SI Dataset S2.

The time-series decomposition separates seasonal changes (COVID-19’s impact on Ramadan), from 2019 to 2020 trend changes (COVID-19’s impact on activity patterns generally). We only use 2019 and 2020 trend means when calculating the percent change in trend radiance from COVID-19, in order to diminish the impact of longer term trends like electrification and urbanization. For the seasonal change in radiance, we subtract the seasonal component (derived from 2015 to 2019 medians) from the detrended 2020 data. For this analysis, any urban area where the seasonal component is not at least two times greater than the remainder component is designated “non-seasonal”, and seasonal change is recorded as zero.

## Supplementary Information

Supplementary Information 1.

Supplementary Information 2.

## Data Availability

All data generated or analysed during this study are included in this published article (and its Supplementary Information files).

## References

[1] Sawaya T, Ballouz T, Zaraket H, Rizk N (2020). Coronavirus disease (covid-19) in the middle east: A call for a unified response. Front. Public Health.

[2] Baloch Z (2020). Unique challenges to control the spread of covid-19 in the middle east. J. Infect. Public Health.

[3] Quadri SA (2020). Covid-19 and religious congregations: Implications for spread of novel pathogens. Int. J. Infect. Dis..

[4] World Health Organization (2021). Safe Ramadan Practices in the Context Of Covid-19: Interim Guidance, 7 April 2021.

[5] Mubarak N (2020). Corona and clergy-the missing link for effective social distancing in Pakistan: Time for some unpopular decisions. Int. J. Infect. Dis..

[6] Ziabari, K. *Sacred Ignorance: Covid-19 reveals Iran Split* (2020). https://asiatimes.com/2020/03/sacred-ignorance-covid-19-reveals-iran-split/ (Accessed 11 January 2021).

[7] Elnahla, N. & Abdo, G. *The Pandemic Tips the Balance Between Mosque and State*. https://www.foreignaffairs.com/articles/middle-east/2020-08-13/pandemic-tips-balance-between-mosque-and-state (Accessed 11 January 2021).

[8] Cheng C, Barceló J, Hartnett AS, Kubinec R, Messerschmidt L (2020). Covid-19 government response event dataset (coronanet v. 1.0). Nat. Hum. Behav..

[9] Zheng Q (2020). Hit-covid, a global database tracking public health interventions to covid-19. Sci. Data.

[10] Hale T (2021). A global panel database of pandemic policies (oxford covid-19 government response tracker). Nat. Hum. Behav..

[11] Ilin C (2021). Public mobility data enables covid-19 forecasting and management at local and global scales. Sci. Rep..

[12] Buckee C, Noor A, Sattenspiel L (2021). Thinking clearly about social aspects of infectious disease transmission. Nature.

[13] Alotaibi N, Almutairi S, Alotaibi M, Alotaibi MM, Alsufian T (2020). The extent of commitment of saudis during holy ramadan to social distancing measures required for the prevention of transmission of covid-19. J. Community Health.

[14] Hanafi, Y. *et al.* Indonesian ulema council fatwa on religious practices during covid-19 pandemic: An investigation of muslim compliance. *Res. Sq. Prepr.* (2020). 10.1007/s10943-022-01639-w PMC9418652 36029452

[15] Al-Hasan A, Yim D, Khuntia J (2020). Citizens’ adherence to covid-19 mitigation recommendations by the government: A 3-country comparative evaluation using web-based cross-sectional survey data. J. Med. Internet Res..

[16] Walsh, D. *As Ramadan Begins, Muslims (Mostly) Accede to Pandemic Orders* (2020). https://www.nytimes.com/2020/04/24/world/middleeast/coronavirus-ramadan-2020.html (Accessed 11 January 2021).

[17] Saleh SN, Lehmann CU, McDonald SA, Basit MA, Medford RJ (2021). Understanding public perception of coronavirus disease 2019 (covid-19) social distancing on twitter. Infect. Control Hosp. Epidemiol..

[18] Google. *Google Covid-19 Community Mobility Reports*. https://www.google.com/covid19/mobility/ (Accessed 9 May 2020).

[19] Apple. *Apple Mobility Trends Reports*. https://covid19.apple.com/mobility (Accessed 09 May 2020).

[20] Levin N (2020). Remote sensing of night lights: A review and an outlook for the future. Remote Sens. Environ..

[21] Román MO, Stokes EC (2015). Holidays in lights: Tracking cultural patterns in demand for energy services. Earth’s Future.

[22] Liu S, Li X, Levin N, Jendryke M (2019). Tracing cultural festival patterns using time-series of viirs monthly products. Remote Sens. Lett..

[23] Levin N, Ali S, Crandall D, Kark S (2019). World heritage in danger: Big data and remote sensing can help protect sites in conflict zones. Glob. Environ. Change.

[24] Stokes, E. C. *et al.* Urban applications of nasa’s black marble product suite. In *2019 Joint Urban Remote Sensing Event (JURSE)*, 1–4 (IEEE, 2019).

[25] Stathakis D, Baltas P (2018). Seasonal population estimates based on night-time lights. Comput. Environ. Urban Syst..

[26] Enenkel M (2019). Emergencies do not stop at night: Advanced analysis of displacement based on satellite-derived nighttime light observations. IBM J. Res. Dev..

[27] Xu G, Xiu T, Li X, Liang X, Jiao L (2021). Lockdown induced night-time light dynamics during the covid-19 epidemic in global megacities. Int. J. Appl. Earth Observ. Geoinf..

[28] Liu Q (2020). Spatiotemporal patterns of covid-19 impact on human activities and environment in mainland china using nighttime light and air quality data. Remote Sens..

[29] Elvidge CD, Ghosh T, Hsu F-C, Zhizhin M, Bazilian M (2020). The dimming of lights in china during the covid-19 pandemic. Remote Sens..

[30] Ghosh T, Elvidge CD, Hsu F-C, Zhizhin M, Bazilian M (2020). The dimming of lights in india during the covid-19 pandemic. Remote Sens..

[31] Stokes EC (2021). Retired satellites: A chance to shed light. Science.

[32] Wang Z (2021). Quantifying uncertainties in nighttime light retrievals from suomi-npp and noaa-20 viirs day/night band data. Remote Sens. Environ..

[33] Wickham H (2016). ggplot2: Elegant Graphics for Data Analysis.

[34] Saudi Arabia to enforce coronavirus curfew during eid (2020). https://www.aljazeera.com/news/2020/5/13/saudi-arabia-to-enforce-coronavirus-curfew-during-eid (Accessed 11 January 2021).

[35] Messner, J. *et al.**The Fragile States Index* (2018). https://fragilestatesindex.org/ (Accessed 05 January 2021).

[36] Ciftci S, Robbins M, Zaytseva S (2021). Devotion at sub-national level: Ramadan, nighttime lights, and religiosity in the Egyptian governorates. Int. J. Public Opin. Res..

[37] Gardaworld. *Turkey: Authorities to Implement 48-h Lockdown in 31 Cities as of April 11/Update 14* (2021). https://www.garda.com/crisis24/news-alerts/331426/turkey-authorities-to-implement-48-hour-lockdown-in-31-cities-as-of-april-11-update-14 (Accessed 11 January 2021).

[38] DuvaR.English. *Turkey Declares Curfew for Weekend in 31 Major Cities* (2020). https://www.duvarenglish.com/coronavirus/2020/04/10/turkey-declares-curfew-for-weekend-in-31-metropolitan-cities (Accessed 24 August 2021).

[39] Ferguson N (2007). Capturing human behaviour. Nature.

[40] Funk S (2015). Nine challenges in incorporating the dynamics of behaviour in infectious diseases models. Epidemics.

[41] Lee M (2020). Human mobility trends during the early stage of the covid-19 pandemic in the United States. PLoS ONE.

[42] Watson OJ (2021). Leveraging community mortality indicators to infer covid-19 mortality and transmission dynamics in Damascus, Syria. Nat. Commun..

[43] Kuechly HU (2012). Aerial survey and spatial analysis of sources of light pollution in Germany. Remote Sens. Environ..

[44] Hale JD (2013). Mapping lightscapes: Spatial patterning of artificial lighting in an urban landscape. PLoS ONE.

[45] Luginbuhl CB, Lockwood GW, Davis DR, Pick K, Selders J (2009). From the ground up I: Light pollution sources in flagstaff, Arizona. Publ. Astron. Soc. Pac..

[46] Karlinsky A, Kobak D (2021). Tracking excess mortality across countries during the covid-19 pandemic with the world mortality dataset. Elife.

[47] Tuysuz, G. *Turkey Enters First National Lockdown as Covid-19 Cases Rise* (2021). https://www.cnn.com/2021/04/29/europe/turkey-enters-national-lockdown-intl/index.html (Accessed 01 November 2021).

[48] ICG. *Pakistan’s Covid-19 Crisis* (2020). https://www.crisisgroup.org/asia/south-asia/pakistan/b162-pakistans-covid-19-crisis (Accessed 11 January 2021).

[49] Hassan, S. R. *Pakistan’s Sindh Province Bans Prayer at Mosques During Ramadan* (2020). https://www.reuters.com/article/us-health-coronavirus-ramadan-pakistan/pakistans-sindh-province-bans-prayer-at-mosques-during-ramadan-idUSKCN226166 (Accessed 11 January 2021).

[50] Geo-News. *Lockdown Restrictions to Stay in Place Throughout Ramadan: Sindh Government* (2020). https://www.geo.tv/latest/285830-lockdown-restrictions-to-stay-in-place-throughout-ramadan-sindh-government (Accessed 11 January 2021).

[51] Looney, R. *Covid-19 in Iran* (2020). https://www.milkenreview.org/articles/covid-19-in-iran (Accessed 11 January 2021).

[52] Behravesh, M. *The Untold Story of How Iran Botched the Coronavirus Pandemic* (2020). https://foreignpolicy.com/2020/03/24/how-iran-botched-coronavirus-pandemic-response/ (Accessed 11 January 2021).

[53] Guthrie, E. & Shapiro, J. *Yemen’s Other War: How Covid-19 is Impacting Life in Yemen* (2020). https://www.premise.com/yemens-other-war-how-covid-19-is-impacting-life-in-yemen/ (Accessed 24 August 2021).

[54] MEE. *Coronavirus: Spared by the Pandemic, Yemenis Enjoy an Unexpectedly Normal Ramadan* (2020). https://www.middleeasteye.net/news/coronavirus-yemen-spared-pandemic-unexpectedly-normal-ramadan (Accessed 11 January 2021).

[55] IOM. *IOM Displacement Tracking Matrix Covid-19 Dashboard Movement Restrictions* (2020). http://iraqdtm.iom.int/COVID19 (Accessed 24 August 2021).

[56] Papadopulos, B. *lockdowns and Curfews are Nothing New on the West Bank* (2020). http://slate.com/news-and-politics/2020/04/for-palestinians-the-coronavirus-lockdown-feels-somewhat-familiar.html (Accessed 24 August 2021).

[57] Freedom-House (2020). Freedom in the world 2020. Civil Libert..

[58] Hasan, H. *Coronavirus in Conflict Zones: A Sobering Landscape* (2020). https://carnegie-mec.org/2020/04/14/iraq-and-coronavirus-pub-81527 (Accessed 11 January 2021).

[59] Maki, D. *Syria is Facing a Covid-19 Catastrophe* (2020). https://www.mei.edu/publications/syria-facing-covid-19-catastrophe (Accessed 11 January 2021).

[60] Mehrl M, Thurner PW (2020). The effect of the covid-19 pandemic on global armed conflict: Early evidence. Polit. Stud. Rev..

[61] Al Jaffal, O. *Implementation of Curfew to Fight Covid-19 Proves Difficult in Iraq. Al-Monitor* (2020).

[62] ICG. *Covid-19 and Conflict: Seven Trends to Watch* (2020). https://www.crisisgroup.org/global/sb4-covid-19-and-conflict-seven-trends-watch (Accessed 11 January 2021).

[63] de Harder, C. *A Polarised Nation During a Global Pandemic: The Libyan Predicament* (2021). https://www.cspps.org/Polarised-Nation-covid19-libya.

[64] Calabrese, J. *Iraq’s Fragile State in the Time of Covid-19* (2020). https://www.mei.edu/publications/iraqs-fragile-state-time-covid-19 (Accessed 07 January 2021).

[65] Dyer, P., Schaider, I. & Letzkus, A. *Infographic: Covid-19 Vaccination Efforts in the Middle East and North Africa* (2021). https://www.brookings.edu/interactives/covid-19-vaccination-efforts-in-the-middle-east-and-north-africa/ (Accessed 11 January 2021).

[66] Prothero RM (1977). Disease and mobility: A neglected factor in epidemiology. Int. J. Epidemiol..

[67] Tatem AJ, Rogers DJ, Hay SI (2006). Global transport networks and infectious disease spread. Adv. Parasitol..

[68] Stoddard ST (2009). The role of human movement in the transmission of vector-borne pathogens. PLoS Negl. Trop. Dis..

[69] Román MO (2018). Nasa’s black marble nighttime lights product suite. Remote Sens. Environ..

[70] Schiavina, M., Moreno-Monroy, A., Maffenini, L. & Veneri, P. *Ghsl-oecd Functional Urban Areas* (Tech. Rep, JRC Technical Report, 2019).

[71] Meyer, D. & Buchta, C. *proxy: Distance and Similarity Measures. R Package Version 0.4-15* (2015).

