# Supplementary material for: Tracking COVID-19 urban activity changes in the Middle East from nighttime lights
Source: Sci Rep. 2022 May 16;12:8096. doi: 10.1038/s41598-022-12211-7 (PMC9109745; doi:10.1038/s41598-022-12211-7)
Supplement: Supplementary file 1 — Supplementary Information 1. [file 41598_2022_12211_MOESM1_ESM.pdf]

# Supplementary Information

## Tracking COVID-19 urban activity changes in the Middle East from nighttime lights

**Eleanor C. Stokes, Miguel O. Román**

**Eleanor C. Stokes.**
**E-mail: estokes@usra.edu**

### This PDF file includes:

Supplementary text
Tables S1 to S4 (not allowed for Brief Reports)
Legends for Dataset S1 to S2
SI References

### Other supplementary materials for this manuscript include the following:

Datasets S1 to S2

## Supporting Information Text

**Description of data availability.** Satellite data supporting this research (VNP46A2) are available from the NASA LAADS DAAC (https://ladsweb.modaps.eosdis.nasa.gov/missions-and-measurements/products/VNP46A2/). Processed data used to produce the results in this study are included as file attachments.

### SI Dataset S1 (FUA.zip)

Nightlights time series data for each FUA for each year are included in the FUA file, with headings as described below:

- SITE: Site ID based on the FUA indexing used in the GHS-FUA dataset (1).
- MONTH, DATE, YEAR: The month, day, and year of NTL observation
- TOA: Top of atmosphere radiance
- L3 DAILY: Lunar BRDF-Adjusted Nighttime Lights Daily Level 3 radiance
- L3 DAILY EXTRAPOLATED: Lunar BRDF-Adjusted Nighttime Lights Daily Level 3 radiance extrapolated so that no data values are ignored
- L3 DAILY 7DAY WINDOW: Level 3 radiance extrapolated and smoothed over a 7 day window
- L3 DAILY 14DAY WINDOW: Level 3 radiance extrapolated and smoothed over a 14 day window
- L3 DAILY 30DAY WINDOW:Level 3 radiance extrapolated and smoothed over a 30 day window
- MOONFRAC: Moon phase fraction (new moon to full moon)
- PERC URB: percent urban pixels within the FUA
- COUNT: number of pixels within the FUA
- %QA LE 1: Percent of pixels within the FUA with a quality flag less than or equal to 1 (high or good quality)
- %QA EQ 2:Percent of pixels within the FUA with a quality flag equal to 2 (poor quality)
- %QA EQ 255:Percent of pixels within the FUA with a quality flag equal to 255 (no retrieval)
- MAJORITY QA FLAG: Quality flag of majority of pixels within the FUA (255, 1 or 0; 2 is considered 255)
- E(X): E as defined in the methods section "nightlights dataset"
- EPSILON(X): $\varepsilon$ as defined in the methods section "nightlights dataset"
- BM AOD: Black Marble aerosol optical depth
- STAT LABEL: High

**SI Dataset S2 (urban_areas_season_general_change.csv;General(trend)andseasonalpercentchangeperurbanarea)**

We provide the analyzed results used to create Figure 6 in the csv file named "urban areas season general change". Headings in this csv are as follows:

- Cntry ISO: Country 3 letter ISO abbreviation
- SITE: Site ID based on the FUA indexing used in the GHS-FUA dataset (1).
- trendperchange: trend (general) percent change
- seasonperchange: season (Ramadan) percent change
- trendchange: NTL radiance trend change
- seasonchange: NTL radiance season change
- trendmean: NTL radiance trend mean
- seasonmean: NTL radiance season mean
- remaindermean: NTL radiance remainder mean
- eFUA name: FUA name
- Cntry name: Country name
- FUA area: FUA land area
- Lon: longitude
- Lat: latitude
- numcits: number of urban areas in specified country

## R Code for drops and falls identification

Type or paste text here. You may break this section up into subheads as needed (e.g., one section on “Materials” and one on “Methods”).

The following R code was used to identify rises and falls in the NTL time series for Figure 3 and Table S1, and was derived from Stack Overflow: https://stackoverflow.com/questions/63226725/find-the-biggest-drops-rises-in-a-time-series-without-a-loop-preferably-using-t

```
rise_and_falls <- function(value, time, gap_width = 5, top = 15, type = "fall") {
  type <- match.arg(type, c("fall", "rise"))
  if (type == "fall") {
    rle <- rle(sign(diff(value)) == -1)
  } else {
    rle <- rle(sign(diff(value)) == 1)
  }
  rle$values <- !rle$values & rle$lengths <= gap_width | rle$values
  rle <- rle(inverse.rle(rle)) # Clean up changed runs
  df <- data.frame(
    start = cumsum(rle$lengths) - rle$lengths + 1,
    end = cumsum(rle$lengths),
    len = rle$lengths,
    drop = rle$values
  )
  df <- transform(
    df,
    start_value = value[start],
    end_value = value[end],
    start_time = time[start],
    end_time = time[end]
  )
  df$diff <- df$start_value - df$end_value
  df <- df[order(df$diff),]
  if (type == "fall") {
    tail(df, top)
  } else {
    head(df, top)
  }
}
```

## Supplementary Tables

**Table S1. Prevalence of Urban Areas in each country with drops in NTL corresponding to physical distancing policies**

| Country | # of UAs | % UAs w/ NTL drop >1% at policy enactment date | % UAs w/ NTL drop >5% at policy enactment date | % of UAs w/ max 2020 NTL drop at policy enactment date |
|---|---|---|---|---|
| Afghanistan | 13 | 54 | 38 | 15 |
| Bahrain | 1 | 100 | 100 | 100 |
| Egypt | 77 | 83 | 73 | 53 |
| Iran | 156 | 76 | 73 | 50 |
| Iraq | 63 | 59 | 40 | 13 |
| Israel | 9 | 89 | 67 | 56 |
| Jordan | 8 | 88 | 75 | 75 |
| Kuwait | 1 | 100 | 100 | 100 |
| Libya | 6 | 0 | 0 | 0 |
| Oman | 8 | 88 | 75 | 13 |
| Pakistan | 39 | 79 | 79 | 56 |
| Palestine | 6 | N/A | N/A | N/A |
| Qatar | 2 | 100 | 100 | 100 |
| Turkey | 116 | 47 | 37 | 25 |
| Saudi Arabia | 39 | 90 | 82 | 62 |
| United Arab Emirates | 6 | 83 | 83 | 50 |
| Yemen | 21 | 38 | 38 | 24 |
| | | | | |

Table statistics are based on the percentage of urban areas that had a disruption in the nightlights time series within 1 week of the onset of a COVID-19 national lockdown or curfew. *No data in database on Syria

**Table S2. Consistency of trend and Ramadan decreases amongst sampled urban areas (UAs) within Middle Eastern countries**

| Country | % UAs w/ sustained general decrease | % UAs w/ Ramadan decrease | # of UAs sampled | # of Seasonal UAs sampled |
|---|---|---|---|---|
| Afghanistan | 85 | 100 | 13 | 7 |
| Bahrain | 0 | 100 | 1 | 1 |
| Egypt | 92 | 100 | 77 | 33 |
| Iran | 47 | 100 | 156 | 1 |
| Iraq | 24 | 33 | 63 | 6 |
| Israel | 22 | NA | 9 | 0 |
| Jordan | 88 | 100 | 8 | 4 |
| Kuwait | 0 | 100 | 1 | 1 |
| Libya | 50 | 50 | 6 | 2 |
| Oman | 75 | 67 | 8 | 3 |
| Pakistan | 72 | 71 | 39 | 7 |
| Palestine | 50 | 100 | 6 | 2 |
| Qatar | 100 | 100 | 2 | 1 |
| Saudi Arabia | 87 | 96 | 39 | 28 |
| Syria | 65 | 100 | 15 | 1 |
| Turkey | 46 | NA | 107 | 0 |
| United Arab Emirates | 50 | 100 | 6 | 3 |
| Yemen | 48 | 54 | 21 | 13 |

**Table S3. Ramadan and general percent change in NTL in urban areas**

| Country | # of UAs | trend $\delta\%$ | stdev | seas. $\delta\%$ | stdev |
|---|---|---|---|---|---|
| Afghanistan | 13 | -16 | 17 | -22 | 35 |
| Bahrain | 1 | 5 | NA | 0 | NA |
| Egypt | 77 | -12 | 8 | -19 | 30 |
| Iran | 157 | -1.5 | 12 | 0 | 0 |
| Iraq | 63 | 8 | 20 | -1 | 9 |
| Israel | 9 | 1 | 3 | 0 | 0 |
| Jordan | 8 | -7 | 9 | -31 | 44 |
| Kuwait | 1 | 2.4 | NA | 0 | NA |
| Libya | 6 | -4 | 9 | 0 | 0 |
| Oman | 8 | -26 | 35 | -4 | 11 |
| Pakistan | 43 | -11 | 30 | -4 | 18 |
| Palestine | 6 | 0 | 5 | -33 | 52 |
| Qatar | 2 | -7 | 7 | -37 | 53 |
| Saudi Arabia | 39 | -16 | 19 | -41 | 41 |
| Syria | 23 | -1 | 23 | -2 | 9 |
| Turkey | 116 | 0 | 7 | 0 | 0 |
| United Arab Emirates | 6 | -4 | 12 | -14 | 24 |
| Yemen | 21 | 6 | 30 | 9 | 42 |

**Table S4. Euclidean distances between z-normalized time series with and without Ramadan**

| City | Country | Euclidean_retail | Euclidan workplace | without Ramadan retail | without Ramadan workplace |
|---|---|---|---|---|---|
| Aswan | Egypt | 7.159283 | 6.997588 | 4.631189 | 4.83738 |
| Beni Suef | Egypt | 14.34316 | 13.44225 | 10.52004 | 9.250136 |
| Damietta | Egypt | 15.43504 | 15.86819 | 12.74098 | 12.57527 |
| Faiyum | Egypt | 15.69915 | 13.32521 | 12.03283 | 9.793277 |
| Luxor | Egypt | 9.682988 | 10.06515 | 5.202898 | 5.757442 |
| Port Said | Egypt | 6.758544 | 6.981101 | 5.355609 | 4.981935 |
| Qena | Egypt | 8.595027 | 8.425544 | 4.399758 | 4.409314 |
| Sohag | Egypt | 8.422177 | 8.739884 | 5.58966 | 6.276906 |
| Ashkelon | Israel | 11.13915 | 11.43072 | 9.027025 | 9.291017 |
| Haifa | Israel | 9.700579 | 9.255399 | 6.64526 | 6.552197 |
| Tel Aviv | Israel | 15.26607 | 14.90429 | 11.38382 | 11.08884 |
| Amman | Jordan | 7.668578 | 7.46766 | 5.382479 | 5.169252 |
| Aqaba | Jordan | 7.43405 | 7.409097 | 5.724446 | 5.750571 |
| Irbid | Jordan | 10.63594 | 11.04533 | 10.44379 | 10.81846 |
| Jerash | Jordan | 10.71207 | 10.77262 | 9.526874 | 9.466191 |
| Madaba | Jordan | 6.014188 | 7.299003 | 4.377968 | 4.900461 |
| Doha | Qatar | 11.32695 | 13.01275 | 5.966046 | 7.040856 |
| Antakya | Turkey | 12.19372 | 12.4447 | 10.40172 | 10.37097 |
| Ceyhan | Turkey | 9.390772 | 9.917773 | 8.339713 | 8.793156 |
| İskenderun | Turkey | 9.337888 | 9.998532 | 5.433286 | 6.033392 |
| Kırıkhan | Turkey | 6.655294 | 7.190045 | 5.070286 | 6.205397 |
| Manavgat | Turkey | 9.280786 | 9.283711 | 8.357054 | 8.359113 |
| Nizip | Turkey | 13.04587 | 13.13911 | 5.556619 | 6.893168 |
| Tarsus | Turkey | 18.21036 | 18.24642 | 16.26557 | 16.0426 |
| country | Bahrain | 12.25859 | 13.92396 | 8.108195 | 9.508409 |
| country | Iraq | 14.78619 | 14.32314 | 11.99523 | 12.00298 |
| country | Kuwait | 9.520518 | 9.684281 | 7.308329 | 7.056178 |
| country | Libya | 14.33701 | 15.82757 | 11.06924 | 11.98529 |
| country | Oman | 5.991707 | 5.720562 | 3.719186 | 3.159174 |
| country | Qatar | 8.843766 | 10.68553 | 3.024328 | 4.312439 |
| country | UAE | 6.077362 | 6.148488 | 4.616799 | 4.692921 |
| country | Yemen | 15.33841 | 18.22618 | 10.03497 | 10.52869 |

## References


1. M Schiavina, A Moreno-Monroy, L Maffenini, P Veneri, Ghsl-oecd functional urban areas, (JRC Technical Report), Technical report (2019).
